# Editorial and Miscellaneous

**Published:** 1863-10

**Authors:** 


### EDITORIAL AND MISCELLANEOUS.

### WORK FOR THE MEDICAL SOCIETIES.

Perhaps the first thing to be done in carrying out the comprehensive methods of observation we indicated last month is, to secure complete and thorough geological surveys of each State or territory, county, town and district—the information thus obtained to be generally diffused, and not left to moulder away among the dusty archives of fossil State libraries. At all events each physician should make it a fundamental point to understand the geology of his own region of country, and, if there is not a record in the central bureau which we propose, he should briefly sketch it in the first "case" which he reports.

What may be termed the *accidental conformation* of the earth's surface also is to be noted. The hills and valleys, the levels and elevations, the sources and direction of running waters, the extent or absence of standing water, alluvium and diluvium, the porosity of the surface at all points, the matters held in solution or suspension, or deposited by the waters.—The character of vegetation, including the complete botanical history of indigenous and exotic growths. And with this the zoology should be allied. Then the meteorology—the dew-point, the times and amount of falling rains and general hygrometric condition. Thermometric and barometric variations—the general direction of winds, and the character of the surfaces over which they have come.

Emanations from the earth, gaseous or organic, from decomposing dead or disintegrating live matter. The effluvium from men or other animals collected together, requiring study of the atmospheric changes produced by their isolation or aggregation. The presence of manufacturing establishments or other exhibitions of human industry, changing, more or less, the air in great or small spaces. Electrical conditions, magnetic conditions, and, in fine, every physical influence operative upon man or animals.

Then the general *physique* of the men who inhabit the country whose peculiarities have been thus sharply scrutinized—whether native or immigrant; their ancestry and nationality; the conditions of their origin and race, and the changed or similar conditions under which they are now placed. The relations of inter-marriages of the races, or the prevalence of marriages of the same race "in and in," albeit out of the limits of illegal consanguinity. The occupation, habits of life, dwellings, clothing, food, including methods of cookery, drinks whether as ordinary beverages or exhilarants. Whether accumulated in masses or scattered. The general or particular mental influences operating them. The ratio of mortality from all causes to the number of living and the number born. The proportion of marriages and births, legitimate and illegitimate. These involving a complete registration of births, marriages, and deaths. Then a registry of diseases of whatever variety or intensity; not of extraordinary or exceptional cases, although these should be put down, but of ordinary and common cases. The duration and relative mortality of each, and all the time with special reference to the general and particular conditions under which each is placed. Among these conditions we may place the medical treatment, and all supposed remedial measures.

The history of Epidemics of every variety should receive particular attention, not, perhaps, because this would tend better to elucidate the great mysteries of disease, but that they may be proved, as has been wisely surmised, to be under the control of orderly laws, and largely, if not wholly preventible. The concentrated effects of epidemics strike the public attention, and awaken public anxiety, beyond the vastly more deadly causes which, operating all the time, sap the foundations of the general health, and, here and there at scattered points, rob of life.

As it is the design of this paper merely to indicate, not to elaborate, methods, it will not be expected that we shall point out everything necessary to be observed. In these days of marvelous developments, when spiritual rappings are believed by some, even of the magnates of the land, to render unnecessary any exercise of the brains, it seems proper for us to recall the professional mind to the strictly Baconian method of arriving at the truth. It is fashionable to believe that it can be secured by a bold *coup d'etat.* There is not one of us but what has built up on the little foundation of what we have seen or think we have seen, divers crude opinions to which we adhere with the most inflexible tenacity. In truth the crude "system" of the thinking man is infinitely better than the *no-system* or bold dogmatism of the unthinking man.

We are ready to admit that Veratrum Viride and Chlorate of Potash are a long way in advance of the blood-letting and salivating of old time—we will even confess that Scutellaria and the Sulphites surpass in intrinsic energy the "oil from a dead man's brains," of mediaeval medicine, or the *Mistura Pythonis* of later times,—but we must still be permitted to say that the use of all these things savors of the baldest empiricism, whilst yet we are ignorant of the diseases themselves, and the *essential relations* of their circumstances. Facts standing isolated are little better than fictions—wrongly interpreted they are worse, for a little truth gives great weight and power to falsehood. A little fragment of mineral may deflect the magnetic needle, or wholly reverse its direction. To get just ideas it is needful to approximate the *all*, not the partial only, yet the minute, the microscopic, and the partial must be studied, else the comprehensive, the gigantic and the general may be misunderstood.

There have been many efforts on the part of members of the profession to rise to the level of widely extended inquiry and research. There are few who will venture to deny its importance—nay, its paramount importance—but, practically, efforts in this direction have generally failed. The only approximation to success has been in the armies of the world, preeminently in our own army, and at the present time, and the fruits of this are not yet gathered.

*Nequaquam nos homines sumus, sed partes hominis. Ex omnibus, aliquid fieri potest, idque non magnum,—ex singulis fere nihil.* Thus wrote Scaliger long since, and the centuries can not contradict it. The greatest of minds accomplishes "almost nothing" single handed, but by union *something* can be done. Associated effort can secure what cannot otherwise be attained. Failures to succeed in the comprehensive inquiries we have indicated, are immensely more numerous than successes, and this notwithstanding the medical societies. Alas, the medical societies resolve themselves into Trades-Unions, Punitive Leagues, and Cerberi over the Code. They would expel somebody who pronounces not the Shibboleth as their ears demand, and who does not believe in pitiful specifics, and pitiful *sine qua nons*—apotheosized by hecatombs of deaths, and, here and there, perchance a cure—perchance an escape.

The difficulty and the remedy are alike obvious. The very persons from whom we most anxiously wish information, are the very ones who are the most overworked in the practice and details of the profession. They have literally no time for elaborate reports or voluminous essays. A thousand men will answer yes or no, where one will write a reply to a general circular of inquiry. And yet to be worth anything their reports must be full and explicit, clear and methodical. Let the medical societies at once discuss upon what points knowledge is needed, (Heaven and Earth both know these are abundant enough!) and reduce the whole to questions, carefully digested, and admitting replies, brief and succinct. Then let each member, and as many others as can be interested, be called upon to make periodical answers. To facilitate this, the questions should be printed with blank spaces for answers.

It would be scarcely more trouble for the physician, however extensive his practice, to fill out and return one of these methodical reports than to make out the papers in a policy of life insurance.

Each medical society should furnish its members with such tables of questions, and require answers. The blanks once filled up, should be referred to a competent bureau or committee, and open to individual study, so that the facts might be collated and compared and the legitimate inductions made.—We should thus in a short space of time have an accumulation of material, for analysis and study, which could not fail to throw a flood of light on the nature of disease, and the influences which beget, foster, control or prevent it. Vain speculations, dogmas, theories, hypotheses and notions would have no place in the true medical science which would emerge from the rubbish and vagabond opinions and fancies under which it has so long been smothered. The rage for specifics and miraculously operating drugs, would be appeased. The Pharmacopœia would be shorn of its huge dimensions, and recourse be had by all true physicians to those methods of cure which, without savoring of the miraculous, would be sure; and having under them the substantial groundwork of reason and common sense, would have to that case the unimpeachable relation of cause and effect. As Bacon promised, we believe this knowledge would yield not meagre, accidental fruits, but generous knots and clusters.

---

*A Manual of Instructions for Enlisting and Discharging Soldiers, with Special Reference to the Medical Examination of Recruits, and the Detection of Disqualifying and Feigned Diseases. By* Roberts Bartholow, A. M., M. D., Ass't Surgeon in charge of McDougall General Hospital, Prof. of Mil. Med. Jurisprudence, Army Medical School. Adopted by the Surgeon-General for Issue to Medical Officers of the Army. Philadelphia, J. B. Lippincott & Co. 1863. 12mo. pp. 276.

This is another of the very convenient manuals which the exigencies of the Military service of the country have brought into being. It is strictly a handbook containing a succinct statement of the subject without any pretence of exhaustion of the various topics adverted to, which must necessarily be left to elaborate and extended treatises. The book is commended to the use of those for whom the author intended it.

We regret that the author did not illustrate more fully the subject *Malingering*, by adducing more examples of the ingenious methods often adopted to escape the "imminent, deadly"—draft, and the arduous duties and dangers of the field. The expedients resorted to often exhibit a shrewdness, sagacity, and tact likely to baffle the most experienced surgeon, and show mental acuteness upon the part of the malingerer which would do honor to a better cause.

---

*Correction.*—An Eastern contemporary, relieving itself, slightly, by another discharge of the fearfully vitiated secretions with which its whole system is contaminated, insists that Dr. R. J. LEVIS, late leading and living editor of the "Reporter," is not, as has been stated, a "Surgeon of Volunteers," but a Volunteer Surgeon to a Military Hospital.

It also observes that Dr. O. C. GIBBS, whose contributions have been for some time missed from the columns of the same hebdomadal, has not discontinued his contributions—but has discontinued his contributions—*temporarily.*

---

*Surgeon-General Hammond.*—A contemporary, deceived by newspaper rumor, states that Surgeon-General Hammond has been removed from his position at the head of the Medical Department of the Army. It is, perhaps, unnecessary to state that this is neither true now, nor likely to be in the future.—His services have been too illustrious, and generally acceptable to the army, for any such inexcusable blunder to be committed.

General Hammond's profound knowledge of Hygiene has been appropriately complimented by the request that he should personally inspect the hospitals and camps of the coast and gulf, in order, if possible, to ameliorate the sanitary condition of those portions of the grand army. There cannot be the slightest doubt that great good will result from his visit.

Our contemporary trusts that his successor may be a man of "common sense." We suggest that if he does have a successor with that useful commodity, there is no shadow of hope that the successor can secure the approval of our contemporary.

The order on which Surgeon-General Hammond was sent South is as follows: "The Sanitary condition of the Department of the South and Gulf requiring special attention and care at this time, it is ordered:—That Surgeon-General Wm. A. Hammond proceed by the first steamer sailing from New York to Hilton Head and Charleston Harbor, thence to Key West and New Orleans. He will establish his Head-quarters in the Department of the Gulf until further orders, giving his special personal attention to the medical branch of the service in that Department and in the Department of the South, securing the adoption of the proper sanitary measures required for the preservation of the health of the armies in those Departments. He will report to the Secretary of War every ten days." Upon which statement our readers are able to build their own opinions.

We are gratified to hear that Surgeon J. K. Barnes, late Medical Inspector, is to have charge of the practical details of the bureau at Washington, during the temporary absence of the Surgeon-General. A better selection could scarcely have been made.

---

*Why Is It?*—That Medical men will persist in mounting hobbies? We have the details of a case where an otherwise reputable physician has gone insane on the subject of persulphate of iron in typhoid fever. He is astounded by the fact that after three or four doses the discharges become perfectly *black*—thus showing the potent eliminating action of the metallic compound. Black and green discharges have been long relied upon as proving the efficiency of Calomel in carrying away from the system "foul secretions."

It is to be supposed that these agents producing the same effect in health, merely proves the truth of the Scriptures: Not that which goeth into a man defileth, but that which cometh out—this defileth a man. The medicine swallowed hath nothing to do with the filthy result. The proof of the pudding is the *Cholera Morbus* which follows it. All of which is not in the Scriptures but tolerably true.

---

*Yellow Fever—Phosphorus.*—M. MELIER recently called the attention of the profession in Paris to a highly interesting and suggestive fact. Yellow fever generally rages in those parts around which the phosphorescence of the sea is most prevalent. He sustains the position assumed by M. Lancereaux that the pathological lesions observed in yellow fever are exactly those of phosphoric poisoning, and that the greater part of those persons who have died from the baneful effects of phosphorus have presented the very condition of the liver considered as characteristic of yellow fever.

---

*Suppurative Hepatitis.*—J. C. CAMERON, M. D., Dep. Insp. Gen., strongly urges, in the *Lond. Lancet*, what may be called exploratory punctures in suspected hepatic suppuration. He insists that the danger is comparatively trivial and that the chances of recovery are vastly above those where nature is trusted to discharge the abscess through the lungs or bowels. He bases his opinions on large experience in the East Indies.

---

*Prof. Blaney.*—Surgeon J. V. Z. BLANEY has been appointed Medical Superintendent of the hospitals of the peninsula. This is a position of great and peculiar responsiblity, and we congratulate the department, and the soldiery, on the sagacity which has selected a gentleman so pre-eminently qualified as a physician, a man, and a humanitarian, for the position. Our only fear for our excellent colleague is, the biblical woe pronounced against those "of whom all men speak well."

*Medical Hobbies.*—We have been put in possession of the *facts* of a recent case of reported cure by the use of an old remedy, re-vamped into a modern hobby. We decline to publish the fact of the patient's death at the time the report was made, because it would do no good. To attack the hobby itself would be folly, because it is already broken-in-wind, and "*its eyes is sot.*" What our correspondent has written, if printed, would prove the little leaven which would leaven a big lump of fermentation—not to be caused to cease even by the *Sulphites.*

---

*Pancoast's Styptic.*—C. C. Jewett, Surg. 16th Mass. Vol., reports to the Surg. Gen. of Mass. that Pancoast's Styptic was found preferable to the persulphate of iron in many of the minor cases of hæmorrhage, inasmuch as it leaves the surface of the stump in a healthy condition, and does not produce the thick incrustation so often objectionable after the application of the iron. He does not attempt to give the *rationale* of its operation, but gives the formula from the recipe of Dr. Brinton, Surg. of Vol., and Lecturer on Surgery in the Washington Military College, viz.: ℞. Carbonate of Potash, ʒj; Castile Soap, ʒij; Alcohol, ℥iv. M.

The good effects of this styptic were peculiarly noticed in a case of hæmorrhage occurring fourteen days after amputation at the shoulder joint, probably from one of the acromion branches. Slight pressure on the subclavian for a short time and this styptic were perfectly successful.

---

*Lindsay & Blakiston.—Catalogue of Publications.*—Our thanks are due to the enterprising publication house of Messrs. Lindsay & Blakiston for their annual catalogue of Medical, Surgical, Dental and Scientific books. The list embraces many of the best works published.

---

*Thin Skinned.*—Medical men ought to be pachydermatous, yet, notwithstanding this palpable truth, we regret to hear that some of our whilome friends do present here and there an attenuation of the cuticular envelope, exceedingly to be deplored. The lightest touch of the lash elicits in them groanings indescribable. We tender the assurances of our extreme sympathy, and suggest that thin-skinned children are particularly liable to be injured by the careless use of edged tools. Will not the ice-man add to his other beneficence a suitable quantity of the crystalized compound from the " basin " to keep his adopted children cool?

---

*Professional Acknowledgements.*—The *Boston Journal* records an instance of grateful remembrance of a physician's services which is a strong argument against the otherwise well sustained doctrine of " total depravity." Dr. ——, of the city of Boston, some years since received a legacy, from a lady, of some twenty-five or thirty thousand dollars, *forty years* after services rendered. The lucky doctor was called upon by the patient's husband to deliver her by forceps, which he accomplished and left the house after a stay of twenty minutes. "Of course no charge was made to a fellow practitioner and the circumstances of the case were consigned to the dusty shelves of past professional experience." The notification to the worthy doctor of the magnificent windfall was so incredible to him, that it required strong persuasion to make him believe it. Who wonders? Reduced to francs this legacy throws far into the shade Mr. Thompson's 100.000, and Prof. Scanzoni's 30.000. It is a curious circumstance in this case, that the physician never saw the patient he had delivered during the whole forty years of her subsequent life. The bequest certainly is voidable on the ground of insanity in the testatrix. The action was not at all in accordance with the ordinary workings of human intellect. If the doctor had been a lawyer and saved the individual a few hundred or thousand dollars, by some quip or quirk of legal legerdemain, we could understand the intense gratitude of the client. Practically, we can understand the sound common philosophy conveyed in the Hibernian's reply to the footpad who presented a modest request for the pecuniary effects of his victim, on pain of untimely exit by "villainous saltpetre," from a blunderbus. "Take my life, but spare all I have got."

A man's life is the small dust of the balance,—his health but a whiff of smoke in his estimation—but touch his bank stock, his real estate, and his greenbacks, and Oh! such gratitude as their preserver to him will receive! It is but the other day that a very clever gentleman of the legal persuasion in this city was the recipient of a present of *ten thousand dollars* for having satisfactorily discharged the duties of a salaried adviser of one of our city railroads for a few years past. We have no doubt that our excellent democratic friend (albeit on the wrong side in the Hopps' case,) richly deserved the *douceur*, but we could not help thinking that if he had been merely a doctor, and had only saved the lives of the entire directory, they would have grumbled at his bill, at $2.00 a visit, sagely insisting that the Apostle of Medical Reform would willingly visit patients, which he could not otherwise get, at fifty cents—street railway fare for any distance inclusive.

Who hath services of plate presented? Railway directors and dyspeptic parsons.

Who hath watches of gold set with diamonds, together with complimentary resolutions? Conductors, hotel clerks, and the "bosses" of machine shops.

Who hath snuff-boxes of precious metals, bedecked with costly gems? The snob who concocts a toothsome julep, or puffs in a newspaper the emphysematous shares of the Humbug and Swindle Bank or Railroad.

The subject grows upon us and we forbear. It is said in the good book, "All that a man hath will he give for his life,"—but it is equally true that nothing which he hath will he give to the man who preserves his life. On the whole the preserving of mean lives in worthless bodies is a thankless business.

